# Exhaustive Search of Correspondences between Multimodal Remote Sensing Images Using Convolutional Neural Network

**DOI:** 10.3390/s22031231

**Published:** 2022-02-06

**Authors:** Mykhail Uss, Benoit Vozel, Vladimir Lukin, Kacem Chehdi

**Affiliations:** 1 Department of Information-Communication Technologies, National Aerospace University, Kharkov 61070, Ukraine; uss@xai.edu.ua (M.U.); lukin@ai.kharkov.com (V.L.); 2 Institut d’Electronique et des Technologies du numéRique, IETR UMR CNRS 6164, University of Rennes 1, 22305 Lannion, France; kacem.chehdi@univ-rennes1.fr

**Keywords:** similarity measure, multimodal images, exhaustive search, deep learning, multiple correspondences

## Abstract

Finding putative correspondences between a pair of images is an important prerequisite for image registration. In complex cases such as multimodal registration, a true match could be less plausible than a false match within a search zone. Under these conditions, it is important to detect all plausible matches. This could be achieved by an exhaustive search using a handcrafted similarity measure (SM, e.g., mutual information). It is promising to replace handcrafted SMs with deep learning ones that offer better performance. However, the latter are not designed for an exhaustive search of all matches but for finding the most plausible one. In this paper, we propose a deep-learning-based solution for exhaustive multiple match search between two images within a predefined search area. We design a computationally efficient convolutional neural network (CNN) that takes as input a template fragment from one image, a search fragment from another image and produces an SM map covering the entire search area in spatial dimensions. This SM map finds multiple plausible matches, locates each match with subpixel accuracy and provides a covariance matrix of localization errors for each match. The proposed CNN is trained with a specially designed loss function that enforces the translation and rotation invariance of the SM map and enables the detection of matches that have no associated ground truth data (e.g., multiple matches for repetitive textures). We validate the approach on multimodal remote sensing images and show that the proposed “area” SM performs better than “point” SM.

## 1. Introduction

Image registration aims at bringing two images acquired in different settings into the same coordinate system [1]. The registration process establishes correspondences between the pixels of the two images and allows their joint analysis, including change detection [2], mosaicking [3], fusion [4] and feature extraction. In the field of remote sensing, the most challenging case is multimodal registration when two images represent different modalities, e.g., optical and radar images [5,6,7].

A widely used registration scheme consists of the following steps: detection of keypoints in two images and search of putative correspondences (PCs) between the keypoints, robust estimation of the geometric transformation between the two images based on the matches found [8,9,10]. Advances in deep learning methods make it possible to improve some or all of the steps in this scheme. As a result, the similarity between image patches has been implemented as a CNN in many publications, including [6,11,12,13]. Another research direction is the automation of keypoint detection and its representation. For example, Georgakis et al. proposed an end-to-end learning framework for keypoint detection and its representation for pose invariant 3D matching of depth images [14]. A similar detection of keypoints and representation by an end-to-end deep learning model is used in [15] to learn both geometrical and semantic correspondences between real-life images. Zhou et al. used shallow pseudo-Siamese CNN to process handcrafted multioriented gradient features and demonstrated improved optical-to-radar image registration quality [16]. Learning-based estimation of the geometric transform from prefound keypoints is implemented in [17] and directly from the images in [18]. Finally, all registration steps can be implemented in an end-to-end trainable way [19].

In this paper, we address one particular element of this registration scheme, namely the exhaustive search of PC between two images. Exhaustive search is justified when the search zone has a reasonable volume. For example, in remote sensing, images acquired at different time instances or by different sensors may be initially registered using the sensor orbital parameter and digital elevation model (DEM) data [6,20]. The remaining matching error can be approximated locally by the translation error with small rotation and scale errors [21,22,23]. The translation errors are limited by the accuracy of the sensor geopositioning and are typically less than 100 pixels [8]. Exhaustive search is useful for matching ground control points between radar and optical images [6]. A similar task arises in computer vision for the stereo matching problem [24]. In this case, the distance to a point is computed by comparing the patches between the left and right rectified images. The search zone along the distance is transformed into a search along the disparity between fixed left fragments and right fragments moving along the horizontal epipolar line. The similarity structure along the disparity coordinate can be computed by an exhaustive search using a patch-matching CNN, as in [11], and may also have false matches for regular textures. If the search zone is too large due to large initial errors, including rotation, scale or translation and 3D viewpoint change, the keypoint approach is beneficial. In complex cases, such as multimodal registration, keypoint detection becomes unreliable, and exhaustive search is preferable.

Exhaustive search is a time-consuming operation because it requires the comparison of a fixed fragment of the template image (TI) with many fragments from the reference image (RI) that cover the desired search zone. For some SMs such as normalized correlation coefficient (NCC) [25] and modality independent neighborhood descriptor (MIND) [26], fast algorithms were proposed for exhaustive search. These handcrafted SMs have been outperformed by deep learning approaches that now consistently demonstrate superior performance in the patch-matching task [13]. However, existing methods are either not designed for exhaustive area search or have limitations that we will discuss below.

The comparison of two local fragments in TI and RI might result in multiple matches. This is for example the case for repetitive patterns (e.g., field structure or urban areas in RS images). It is possible that the true match is less likely than some of the false matches. This problem becomes more significant for larger search zones and complex registration cases when the true correspondence might be weak. It is interesting to note that in nonlocal filtering [27], false matches are useful information about long-range correlations of image structures. Here, we assume that exhaustive search should provide all plausible matches within the search zone that can be used in one-to-many matching methods such as multiple-correspondence RANSAC [28]. This requirement is naturally satisfied for handcrafted SMs (such as NCC) when each point in the search space is computed independently from the other points. The same is true when an exhaustive search is performed using patch-matching CNNs designed to compare patches and return a single SM value characterizing their similarity. This approach is computationally inefficient and is overcome by designing CNNs that compute SM values for the entire search zone at once [12,29,30]. In this approach, the output SM values become dependent, and the multiple match detection requirement is not satisfied. Let us consider several examples.

Merkle et al. proposed to consider optical and SAR image matching as a multiclass classification problem, with each class corresponding to a specific shift between TI and RI [29]. In this approach, the patch-matching CNN is trained to predict a heatmap resembling a Gaussian function centered on the true match location. Multiple matches are not considered by design, and their detection quality is not assured. A similar idea is used in [30], except that the 2D Kronecker delta function is employed as the ground truth. Likewise, the authors of [12] proposed a CNN for stereo matching that takes as input the left patch and the right patch covering the entire search zone along disparity and outputs the similarity for each disparity value. The CNN is designed to predict a smooth target distribution centered around the ground truth (GT) disparity value. A similar heatmap approach with a focus only on the main peak is adopted in [16]. In these papers, false matches are considered to be a drawback, and special measures are taken to remove them. Furthermore, the softmax operation is often used as the output layer of the CNN, which makes the magnitude of the main peak of predicted heatmaps dependent on the number and strength of false matches. This dependence complicates the thresholding of the heatmaps to discriminate between true and false correspondences. Another drawback is that these CNNs need to be retrained when the search zone is changed.

In this paper, we address these problems and propose a loss function for training CNN for exhaustive search of multiple matches between two images. The main challenge in solving this task is that GT data exist only for the true correspondence. False matches between RI and TI can be found algorithmically by applying existing SMs, but in this case, the new SM trained with annotated data will be limited by the quality of the reference SM. Annotation of multiple matches by humans is not realistic. To overcome this difficulty, we use only true GT correspondences in the training and add spatial and rotation invariance constraints to the proposed loss function. We show that forcing translation invariance on the predicted SM map results in learning multiple matches within the search zone, including both true and false ones.

The proposed CNN produces an SM map of the size of the search zone. For each pixel, the SM contains the translation vector that points to the closest match and the covariance matrix of the error of this translation vector. Postprocessing of the SM map allows finding multiple matches, localizing them with subpixel accuracy and assigning a score to each of them. Unlike existing methods, the score value does not depend on the number of matches but only on the similarity between RI and TI. An additional benefit is that the SM maps predicted by our method can be tiled to cover a wide search zone without retraining the CNN.

The remaining part of the paper is organized as follows. In Section 2, we first state the problem of training a similarity measure capable of detecting multiple matches between input images. We then introduce the multiterm loss function for training the proposed CNN and explain the meaning of each term. In Section 3, we discuss the experimental results that demonstrate the ability of the proposed SM to detect multiple matches, discriminate between true and false matches and localize the position of each match. We also study the structure of the predicted SM map in comparison with existing methods and illustrate the detected false matches. Finally, in Section 4, we summarize our findings and make some remarks about future work.

## 2. Learned Similarity Measure for Multimatch Case

### 2.1. SM Map Structure and Translation Invariance Property

To design CNN for exhaustive similarity search, we build on our previous work [31], which proposed an SM capable of jointly discriminating and localizing PCs. The proposed SM, called deep localization and similarity measure (DLSM), takes as input two 32 by 32 pixel fragments and provides as outputs the predicted translation vector between these fragments and the covariance matrix of the translation vector prediction error. The determinant of the predicted covariance matrix serves as the SM value and is used to discriminate true and false matches. In this work, we seek an SM with a similar structure but extended from the original processing of a single point in the search space to processing the entire search area at once. In what follows, we will refer to this new SM as DLSMarea or “area” SM as opposed to “point” SM.

The “area” SM differs from the “point” SM in a way that can be illustrated by the requirements applicable to these SM variants. The requirements that apply to a “point” SM are listed below:

- Discrimination between the true and false matches;
- Subpixel accuracy of the true correspondence localization;
- Estimation of the accuracy of the match location, including anisotropic case;
- An “area” SM has additional requirements;
- Translation invariance;
- Plausible multiple match detection;
- Localization and localization accuracy characterizations for all detected matches.

Among these additional requirements the first one, related to translation invariance, needs to be explained. It simply means that the SM map of a large search area can be composed by stacking SM maps calculated for smaller search zones. Translation invariance is naturally satisfied for handcrafted SMs, because the SM values are calculated independently (e.g., NCC, MI [32] or SIFT [33]), and their tiling is trivial. However, if many SM values are jointly predicted by a CNN, as in the case of the proposed SM, their values become mutually dependent and translation invariance is not necessarily satisfied.

Our main observation is that after ensuring translation invariance of the “area” SM in a special way, this CNN automatically learns to detect all plausible correspondences within the search area. We describe this idea in detail in the subsection “loss function”.

### 2.2. DLSMarea Structure

**Figure 1:**
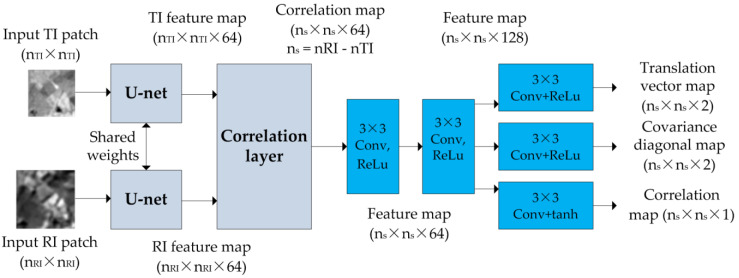
DLSMarea CNN structure.

The DLSMarea structure uses the idea of the correlation layer proposed in [34]. The proposed CNN (Figure 1) takes as input the TI fragment of size $n_{\mathrm{TI}}\times n_{\mathrm{TI}}$ pixels and RI fragment of size $n_{\mathrm{RI}}\times n_{\mathrm{RI}}$ pixels and outputs the SM value in the search zone of size $n_{s}\times n_{s}$ pixels, where $n_{s}=n_{\mathrm{RI}}-n_{\mathrm{TI}}+1$. Both RI and TI fragments are transformed to feature maps of size $n_{\mathrm{TI}}\times n_{\mathrm{TI}}\times n_{F}$ and $n_{\mathrm{RI}}\times n_{\mathrm{RI}}\times n_{F}$ using U-net CNN [35] with weights shared between the RI and TI branches. The correlation layer is implemented as a convolution operation applied to each feature channel, with TI features serving as a convolution filter. The correlation layer produces a correlation map of size $n_{s}\times n_{s}$ pixels. In total $n_{F}$ correlation maps are formed. The architecture allows a search zone of size $n_{s}=8k+1$, where k is positive integer: $n_{s}=17,25,33,41,\ldots$ We denote DLSMarea with a given $n_{s}$ as DLSMarea [$n_{s}$], e.g., DLSMarea17 for $n_{s}=17$.

The correlation maps are processed by two additional convolution layers Conv1 and Conv2. The resulting feature map is transformed into a translation vector map, a covariance matrix diagonal value map and a correlation coefficient map. No output activation is used for the translation vector, “ReLu” activation is used for covariation diagonal values and “tanh” for correlation values. Each pixels of the output SM map represents the stacked values of the translation vector, $t=(\Delta_{x},\;\Delta_{y})$, the diagonal values of the covariance matrix $\sigma_{x}$ and $\sigma_{y}$ and the correlation coefficient $k_{xy}$, i.e., a five-element vector $t=(\Delta_{x},\;\Delta_{y},\sigma_{x},\sigma_{y},k_{xy})$. The CNN outputs have dimension $n_{s}\times n_{s}\times 5$ and contain an SM description for each translation between TI and RI (integer translation). This output is then processed to obtain the subpixel coordinates of all putative correspondences, the covariance matrix of localization error for the correspondence and the similarity value for each match.

### 2.3. Loss Function

**Figure 2:**
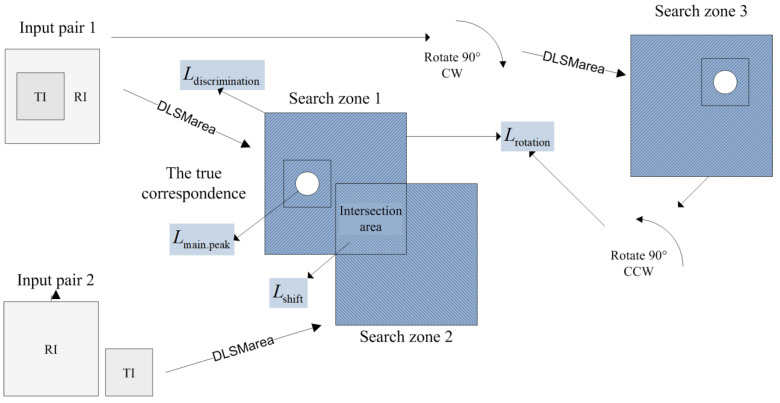
Illustration of the proposed loss function terms.

The training data for the proposed “area” SM are pairs of registered multimodal images. By cropping a random fragment from TI and the corresponding fragments from RI, a training sample is obtained. For each sample, the only GT information is the coordinates of the true correspondence. As discussed above, false matches between TI and RI fragments cannot be annotated or found reliably and automatically. To enable the detection of all plausible matches and satisfy the requirements of the SM map, we propose the following loss function with four terms:

(1) $$L=L_{\mathrm{main}.\mathrm{peak}}+\lambda L_{\mathrm{discrimination}}+\mu L_{\mathrm{shift}}+\nu L_{\mathrm{rotation}},$$

where $L_{\mathrm{main}.\mathrm{peak}}$ is the loss term applied to the SM peak corresponding to the true match (main peak), $L_{\mathrm{discrimination}}$ is the discrimination loss, $L_{\mathrm{shift}}$ is the translation invariance loss term and $L_{\mathrm{rotation}}$ is the additional loss term that helps characterize the localization accuracy for anisotropic textures, λ, μ and ν are hyperparameters. The relationships between the loss terms and the requirements of the “area” SM are described in Table 1 and illustrated in Figure 2. Let us discuss each loss term more in detail.

#### 2.3.1. Main Peak Term

The loss term for the main peak aims at solving the problem of regression with uncertainty. For each pixel of the SM map, the translation vector $t=(\Delta_{x},\;\Delta_{y})$ and the covariance matrix $C=\left[\begin{array}{cc} \sigma_{x}^{2} & \sigma_{x}\sigma_{y}k_{xy}\\ \sigma_{x}\sigma_{y}k_{xy} & \sigma_{y}^{2} \end{array}\right]$ of the estimation error of the translation vector are learned by minimizing the function [31]:

(2) $$\begin{array}{l} L_{\mathrm{main}.\mathrm{peak}}\left(x,y\right)=(\Delta_{x},\;\Delta_{y})\cdot C^{-1}{\left(\Delta_{x},\;\Delta_{y}\right)}^{T}+\ln\left(\left|C\right|\right)=\\ \;\;\;\;\frac{\left[\Delta_{x}^{2}\sigma_{y}^{2}+\Delta_{y}^{2}\sigma_{x}^{2}-2\Delta_{x}\Delta_{y}\sigma_{x}\sigma_{y}k_{xy}^{2}\right]}{\sigma_{x}^{2}\sigma_{y}^{2}\left(1-k_{xy}^{2}\right)}+2\ln\left(\sigma_{x}\right)+2\ln\left(\sigma_{y}\right)+\ln\left(1-k_{xy}^{2}\right)=\\ \;\;\;\;\frac{1}{1-k_{xy}^{2}}\left[\frac{\Delta_{x}^{2}}{\sigma_{x}^{2}}+\frac{\Delta_{y}^{2}}{\sigma_{y}^{2}}-2k_{xy}^{2}\frac{\Delta_{x}}{\sigma_{x}}\frac{\Delta_{y}}{\sigma_{y}}\right]+2\ln\left(\sigma_{x}\right)+2\ln\left(\sigma_{y}\right)+\ln\left(1-k_{xy}^{2}\right). \end{array}$$

Here $\sigma_{x}$, $\sigma_{y}$ and $k_{xy}$ are the components of the covariance matrix predicted by the respective branches of DLSMarea.

#### 2.3.2. Translation Invariance Term

To enforce the translation invariance property, we feed to the second Siamese branch a shifted version of the RI fragment while keeping the same TI fragment. By shifting the RI fragment by (Δx,Δy) pixels, the search zone will be shifted by the same vector.

Ideally, the outputs of DLSMarea that are in the intersecting parts of the search zone for the first and second branches should coincide (see Figure 2 for illustration). This requirement is formalized by the loss term $L_{\mathrm{shift}}$:

(3) $$L_{\mathrm{shift}}=\frac{1}{N}\sum_{\Omega_{xy}}\left(SM_{1}(x,y)-SM_{2}(x+\Delta x,y+\Delta y)\right).$$

where $SM_{1}$ and $SM_{2}$ are SM maps for two branches, and $\Omega_{xy}$ a is set of coordinates where $SM_{1}$ and $SM_{2}$ maps intersect.

By a special selection of the shift vector (Δx,Δy), the loss term (3) could allow the detection of secondary SM peaks in addition to the main one. If the main peak is within the search zone for both Siamese branches, there is a trivial solution that minimizes the loss (3): a single SM peak with a constant value outside this peak (illustration). However, if the shift vector is selected such that the main peak for Branch 1 is outside the search zone for Branch 2, the trivial solution is no longer applicable. Branch 1 must detect both the main peak and any other false matches that might be detected by Branch 2. Minimizing the loss term (3) with a correctly selected shift vector will lead to secondary peaks detection, as well as translation invariance.

#### 2.3.3. Discrimination Loss Term

The discrimination loss is implemented as a binary $L_{2}$ loss applied to the averaged SM values within the main peak mask, $SM_{+}$, and outside this mask, $SM_{-}$. The main peak mask is a neighborhood of ±3 pixels around the location of the true match. The SM values are transformed by the softmax operator prior to $L_{2}$ calculation

(4) $$L_{\mathrm{discrimination}}={\left(0-s_{+}\right)}^{2}+{\left(1-s_{-}\right)}^{2}=2s_{+}^{2},$$

where $s_{+}=\frac{e^{SM_{+}}}{e^{SM_{+}}+e^{SM_{-}}}$, $s_{-}=1-s_{+}$.

#### 2.3.4. Rotation Loss Term

Due to the lack of ground truth value, the covariance matrix of localization errors is difficult to estimate. To stabilize the training, the following information can be used. If registered images are both rotated by an angle α, the covariance matrix $C=\left(\begin{array}{cc} a & c\\ c & b \end{array}\right)$ is transformed into:

(5) $$\begin{array}{l} C\left(\alpha \right)=\left(\begin{array}{cc} \cos\left(\alpha \right) & -\sin\left(\alpha \right)\\ \sin\left(\alpha \right) & \cos\left(\alpha \right) \end{array}\right)C\left(\begin{array}{cc} \cos\left(\alpha \right) & \sin\left(\alpha \right)\\ -\sin\left(\alpha \right) & \cos\left(\alpha \right) \end{array}\right)=\\ =\left(\begin{array}{cc} a\cdot \cos^{2}\left(\alpha \right)+b\cdot \sin^{2}\left(\alpha \right)-2c\cdot \cos\left(\alpha \right)\sin\left(\alpha \right) & \left(a-b\right)\cdot \cos\left(\alpha \right)\sin\left(\alpha \right)+c\cdot \left(\cos^{2}\left(\alpha \right)-\sin^{2}\left(\alpha \right)\right)\\ \left(a-b\right)\cdot \cos\left(\alpha \right)\sin\left(\alpha \right)+c\cdot \left(\cos^{2}\left(\alpha \right)-\sin^{2}\left(\alpha \right)\right) & a\cdot \sin^{2}\left(\alpha \right)+b\cdot \cos^{2}\left(\alpha \right)+2c\cdot \cos\left(\alpha \right)\sin\left(\alpha \right) \end{array}\right) \end{array}$$

Condition (5) should be satisfied only if the CNN receives the same input data independently of α. This is possible for rotation angles $\alpha =\pm k\frac{\pi }{2}$, when the rotated image is obtained by reordering the pixels of the original image. Consequently, we use only one rotation angle $\alpha =\frac{\pi }{2}$. For this, the transformation of the covariance matrix is simplified to:

(6) $$C\left(\alpha \right)=\left(\begin{array}{cc} b & -c\\ -c & a \end{array}\right).$$

To impose the constraint (6), we use both the original and rotated pairs of the CNN input (see Figure 2 for illustration). The output for the rotated pair is rotated backwards by $-\frac{\pi }{2}$. According to (6), after rotation, the values of $\sigma_{x}$ and $\sigma_{y}$ must be swapped and the sign of $k_{xy}$ reversed to match the prediction for the original pair. In mathematical terms, the SM maps ${\left(t_{x},t_{y},\sigma_{x},\sigma_{y},k_{xy}\right)}_{\mathrm{original}}$ and ${\left(t_{x},t_{y},\sigma_{y},\sigma_{x},-k_{xy}\right)}_{\mathrm{rotated}}$ must be equal, where lower subscripts “original” and “rotated” indicate the DLSMarea prediction for the original and rotated pair of frames. To enforce this condition, we use the following loss term:

(7) $$L_{shift}=\left\|{\left(t_{x},t_{y},\sigma_{x},\sigma_{y},k_{xy}\right)}_{\mathrm{original}}-{\left(t_{x},t_{y},\sigma_{y},\sigma_{x},-k_{xy}\right)}_{\mathrm{rotated}}\right\|.$$

### 2.4. Multiple-Correspondence Detection

Putative matches are extracted from the SM map using a three-step algorithm. In the first step, the map of SM values is calculated as $SM_{\det}=\sqrt{\left|C\right|}=\sigma_{x}\sigma_{y}\sqrt{1-k_{xy}^{2}}$. Then, the positions of the PCs are detected as arguments of the local $SM_{\det}$ minima. Redundant detections are filtered out by the nonmaximum suppression (NMS) algorithm. Finally, the position of each PC is refined using the pixels of the neighboring SM map using the algorithm proposed in [31]. This procedure yields the list of detected PCs, the SM value for each PC, the subpixel translation vector between RI and TI for each PC, and the covariance matrix of the translation vector for each PC.

### 2.5. Training Details

For the experiments with DLSMarea, we set $n_{\mathrm{TI}}=32$ and $n_{F}=64$. These particular values were selected as a compromise between processing time and model quality. We provide additional details in Section 3.2 and Section 3.3.

For training, we use pairs of registered multimodal images (see Experimental Section (Section 3) for a description of the training and test datasets). A pair of TI and two RI fragments are cut from a random location of a random image pair. The TI fragment has a size of 32 by 32 pixels. The first RI fragment has a size of $\left(32+n_{s}-1\right)\times \left(32+n_{s}-1\right)$ and is shifted from TI such that the true PC is within the search zone. The second RI fragment has the same size but is shifted from TI such that the true PC is outside the search zone and search zones intersect between selected RI fragments. The third TI–RI pair is formed by rotating the first pair by 90°. This group of three RI–TI pairs forms one sample for the training of DLSMarea.

The proposed DLSMarea is trained with Adam optimizer [36], with a learning rate of 10^−4^ and a decay of 10^−5^. The training takes 500 epochs, with each epoch consisting of 5000 steps. The batch size is set to 32. Hyperparameters are set as λ=1, μ=ν=5. This particular selection was found during a limited-scale grid search for the selected DLSMarea architecture, with a TI size of 32 by 32 pixels and $n_{F}=64$. It leads to an appropriate balance of all loss terms, a better performance of “area” DLSM than “point” DLSM and convergence for all search zone sizes. We also tested that these hyperparameters work well for TIs of size 64 by 64 pixels and $n_{F}=32$. However, we cannot state that these are optimal parameters, and further optimization is reasonable, especially for other choices of CNN structure or parameters.

## 3. Experimental Section

In this experimental section, we aim at validating the main properties of the proposed DLSMarea approach, including its discriminative power, correspondence localization accuracy, multiple match detection capability, translation invariance and computational complexity.

The DLSMarea training is based on 18 registered pairs of multimodal images that were previously used for DLSM training in [31]. These pairs cover visible-to-infrared, optical-to-radar, optical-to-DEM and radar-to-DEM cases. We use the term “optical” for both visual and infrared modalities. In the following, we group all these cases together and define this as the general case. Data for the optical modality are from the Sentinel 2, Landsat 8 and Hyperion platforms, data for the radar modality are from the SIR-C and Sentinel 1 platforms and DEMs are from ASTER Global DEM 2 and ALOS World 30m global DEMs. The image areas corresponding to the four modality pairs are in the following proportions: 75% for visible-to-infrared, 9% for optical-to-radar, 8% for optical-to-DEM and 8% for radar-to-DEM. A total of 4,000,000 patch pairs and the corresponding RI fragments were collected from the above-mentioned registration cases. The size of the RI fragment depends on the $n_{s}$ parameter. These pairs were randomly assigned to the training (75%) and validation (25%) sets.

The test data were collected from another set of 16 registered multimodal pairs covering the same registration cases (see [31,37] for a detailed description). In the current study, the RI fragments have a different size that was not taken into account when collecting the original test set. Therefore, we regenerated the test by collecting a total of 60,000 patch pairs with 50% similar and 50% dissimilar pairs. The modality pairs are equally represented in the test set.

### 3.1. SM Spatial Properties: Tiling

**Figure 3:**
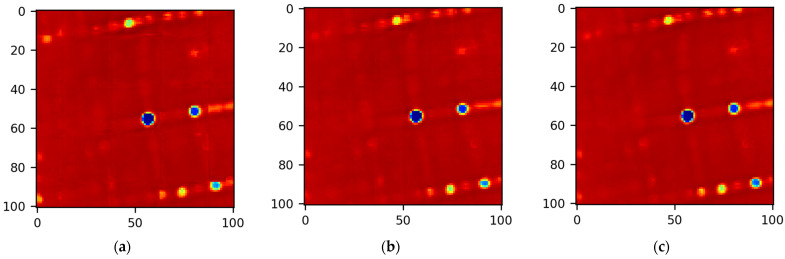
SM map tiling of 100 by 100 pixels using 25 by 25 maps with different overlapping: (**a**) nonoverlapping; (**b**) overlapping with a step of 12 pixels; (**c**) overlapping with a step of 1 pixel.

The “area” SM covering a larger search zone takes more time to train and is redundant in situations where the intended search zone is small. Ideally, a larger search zone can be covered by tiling SM maps calculated for smaller parts of the search zone. This type of nonoverlapping tiling is most likely to result in translation invariance errors. Training DLSMarea with the $L_{\mathrm{shift}}$ loss reduces this source of error but cannot eliminate it completely. The blocking effect can be reduced by means of overlapping tiling with a moving average of SM maps. The best results are expected from an overlapping step of one pixel. However, this variant is not practical due to its high computational complexity. Illustrations of the SM blocking effect are given in Figure 3, where nonoverlapping, overlapping with tiling steps of 12 and 1 pixels are shown. For nonoverlapping tiling, the blocking effect is noticeable but disappears for overlapping tiling (compare Figure 3a with Figure 3b,c). Let us evaluate overlapping steps sufficient for constructing large SM maps from partial maps.

First of all, we observed that the proposed “area” SM has the largest error at the boundary of the output SM map due to the boundary effects of CNN convolutions. Therefore, we discard the 1 pixel layer from the SM map (for example, a 25 by 25 pixel SM map becomes a 23 by 23 pixel map after discarding 1 pixel layer). Since the true SM map does not exist, we use the map obtained with a 1 pixel tiling as the reference one. The other maps are compared to the reference map using the standard deviation (SD) of the relative error: $e_{tiling}=SD\left(SM_{\mathrm{value}}/SM_{\mathrm{value}.\mathrm{ref}}-1\right)$.

**Figure 4:**
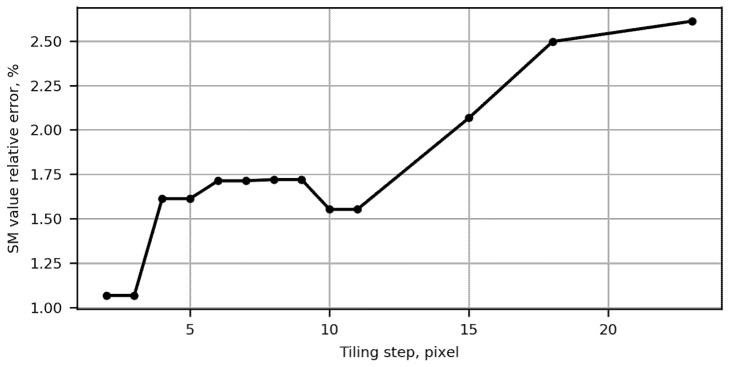
Proposed “area” SM value tiling error.

The dependence of $e_{tiling}$ on the tiling step is shown in Figure 4. For nonoverlapping tiling (right part of the plot), the relative error takes on the largest value of about 2.6%. The relative error has the tendency to decrease monotonically with a reduction of the tiling step. Selecting a tiling with a step close to half the size of the SM map (e.g., 7 or 12 pixels for an SM map of 25 by 25 pixels) decreases the relative error by about twofold (to 1.5%), removes visual blocking artifacts and allows detecting putative correspondences at the edges of SM map blocks. We chose this tiling scheme for all future experiments.

### 3.2. Computational Efficiency

**Figure 5:**
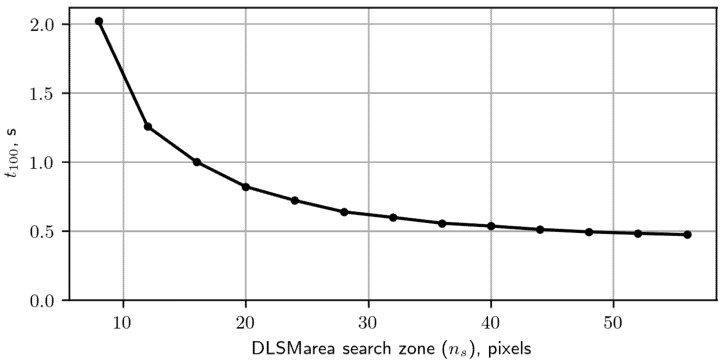
Time for processing search zone ±100 pixels using DLSMarea with different sizes of search zone ($n_{s}$).

Let us compare the computational efficiency of the proposed “area” SM with the “point” approach. We characterize each variant in terms of the inference time required to cover the ±100 pixel search zone with an overlap ratio of 0.5 denoted as $t_{100}$. The experiment was performed with the NVIDIA GeForce GTX 960M GPU. Results are shown in Figure 5.

The time for processing the ±100 pixel search zone decreases as the search zone covered by DLSMarea increases. The first reason is that the complexity of extracting features from the TI image does not depend on the DLSMarea search zone area. The second reason is that for a larger DLSMarea search zone area, the calculations are more efficiently parallelized on the GPU. The time $t_{100}$ decreases fast for $n_{s}$ up to 60 pixels. A further increase of $n_{s}$ does not provide any time savings. Therefore, we limited the next analysis by $n_{s}$, taking the following set of values allowed by the DLSMarea architecture: 17, 25, 33, 41, 49 and 57.

To consider DLSMarea from a practical perspective, we calculate the time required to obtain PCs between two images of size 1000 by 1000 pixels. An image of this size can be covered by approximately 1000 nonoverlapping TI fragments (32 by 32 pixels), yielding a processing time of 1000×0.5s=500s. This time can be further reduced using more powerful GPUs or by performing parallel computations on multiple GPUs. Parallel implementation of SIFT [38] such as CUDA-SIFT can run at 20 frames per second on images with a similar resolution of 1280 by 960 pixels [39]. Compared to NCC, DLSMarea is at least $n_{F}$ times slower (its architecture involves $n_{F}$ convolutions with similar processing complexity as NCC). Nevertheless, the processing time provided by DLSMarea allows large images to be registered in a realistic time and with significantly higher quality, as we will show in the next subsection. The possible scenario for processing a large number of images is the use of a two-stage process: the first stage applies a fast classical algorithm and identifies the failure cases that are processed in the second stage by a “heavy” algorithm like the proposed DLSMarea.

The processing time depends on the parameters of DLSMarea. The dependence on the number of correlation layers $n_{F}$ is linear and the dependence on the TI size is quadratic. The latter is the most important dependence and is caused by the correlation layer convolving with a kernel size equal to the TI size. If the TI size is larger than 32 pixels (e.g., 64 or 128 pixels), the training and inference times increase unreasonably; therefore, we restricted the experimental part to a single TI size value. The reduction of $n_{F}$ improves the inference time but at the expense of the quality of DLSMarea performance. We provide the results for $n_{F}=32$ in the next subsection.

### 3.3. AUC Analysis

The ability of the DLSMarea variants to discriminate between true and false matches is characterized by the area under the curve (AUC) values. The results for NCC, MI, SIFT-OCT, MIND and the “point” and “area” versions with different values of $n_{s}$ are given in Table 2.

Handcrafted methods perform significantly worse than learning-based ones. It is interesting to note that all DLSMarea versions perform better than the “point” version with an AUC gain from 0.3 to 2.8%. The best result is obtained for DLSMarea33 with an AUC value of 86.87% in the general case compared to 84.07 for the “point” DLSM. We attribute this significant gain to the use of the whole RI search zone during training instead of the use of a single point in the search zone for the “point” SM. This effect is similar to the “central-surround” patch-matching approach [40]. There is no clear dependence of the AUC of DLSMarea on $n_{s}$; it varies randomly with $n_{s}$. These fluctuations may be caused by the training process, and we plan to address this issue in future studies.

All results in Table 2 are obtained for $n_{F}=64$ correlation channels. To test the influence of $n_{F}$ on the AUC, we trained the DLSMarea33 version with $n_{F}=32$ and obtained an AUC = 84%. This result is lower than the 86.87% obtained for the version with $n_{F}=64$.

### 3.4. SM Map Comparative Analysis

**Figure 6:**
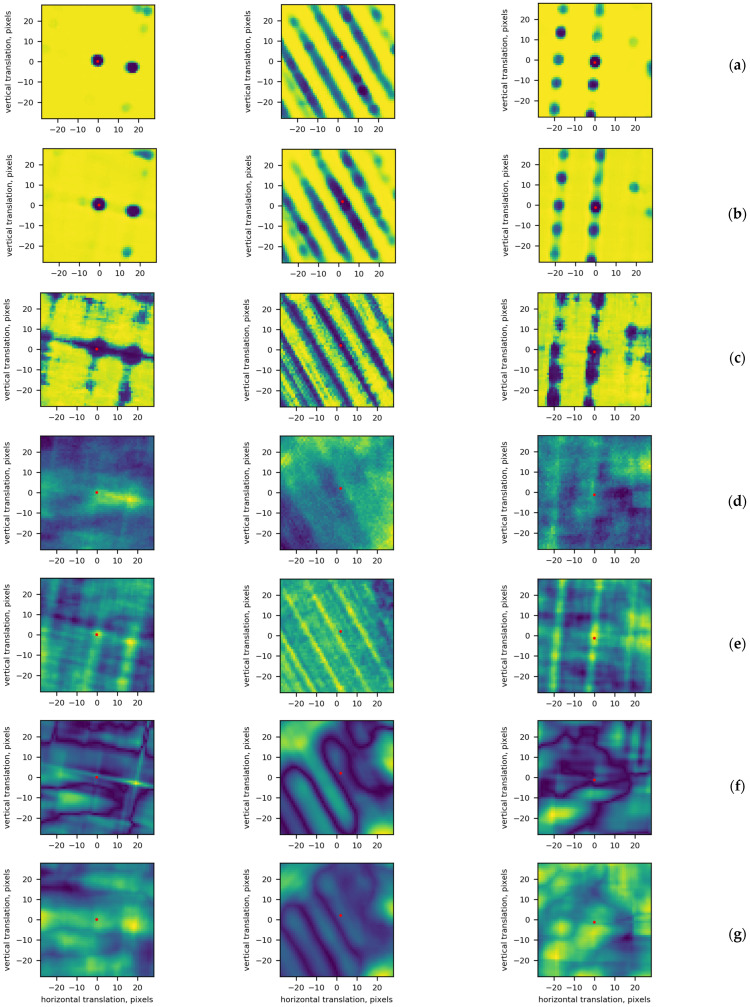
Examples of SM maps calculated for DLSMarea17 (**a**), DLSMarea25 (**b**), original DLSM (**c**), MI (**d**), MIND (**e**), NCC (**f**) and SIFT-OCT (**g**). For DLSMarea and DLSM, blue color means higher similarity; for the rest of the SMs, yellow color means higher similarity. Three columns represent different types of SM maps.

Next, we will compare the appearance of the SM map for several handcrafted SMs and the proposed “area” SM. For the handcrafted SMs, the map is computed on a pixel-by-pixel basis, i.e., by calculating the SM value for each shift vector within the search zone. For this experiment, we set the size of the search zone to ±28 pixels. The SM map produced by DLSMarea is compared with those ones obtained with NCC, MI, SIFT-OCT [33] and MIND similarity measures. For the SIFT-OCT and MIND measures, the value of an SM is calculated as the distance between the descriptors computed for the TI fragment and RI fragments that cover the search zone. The corresponding SM maps are presented in Figure 6.

The three columns of Figure 6 represent three typical situations: localized two-dimensional matching in the first column, matching for a linear structure in the second column and repetitive matching for a regular texture in the third column. The maps in rows (a) and (b) compare results for DLSMarea17 and DLSMarea25. It can be seen that the maps are similar, validating that the training of DLSMarea is stable and does not depend on the size of the search area. The SM maps for “point” DLSM contain more noise and have less distinctive SM map “peaks”. The MIND descriptor has the best performance under multimodal conditions according to our previous study [37]. The SM map produced by this description in row (d) resembles the most results of DLSMarea. The similar composition of the SM extrema for DLSMarea, “point” DLSM and MIND validates that the proposed loss function for training DLSMarea detects both the true and false matches (MIND detects all matches independently). Compared to MIND, DLSMarea’s SM maps are less noisy, locate matches better and have a more uniform background. The SM maps for NCC, MI and SIFT-OCT reveal a similar structure but with less clear details.

**Figure 7:**
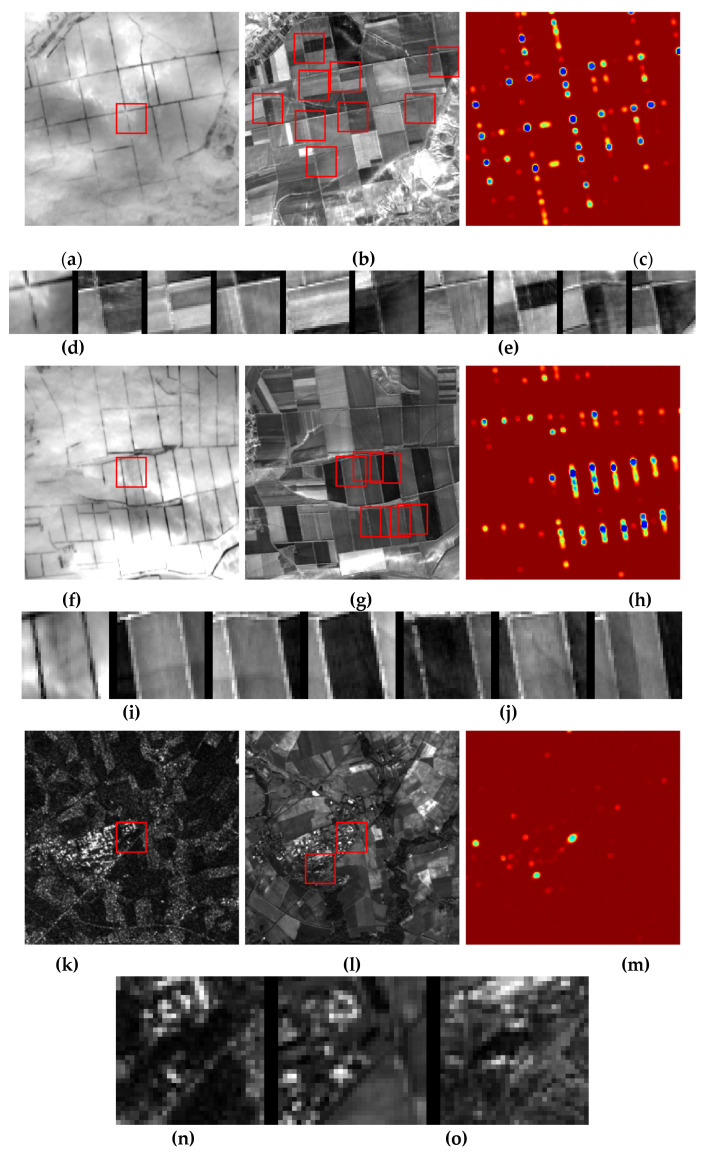
Examples of SM maps and matches found. TI fragment covering the search zone with the part to be searched is marked by a red square (**a**,**f**,**k**), RI fragment covering the search zone where found matches are is marked by red squares (**b**,**g**,**l**), SM map (**c**,**h**,**m**) with red color corresponding to a low similarity and blue color to a high similarity, TI fragment (**d**,**i**,**n**) and RI fragments found (**e**,**j**,**o**), The leftmost RI fragment corresponds to the true correspondence; the following fragments are plausible false matches.

The amount and composition of false matches depend on the content of the registered images. Let us illustrate the type of matches that DLSMarea is able to detect. Figure 7 shows three examples with two first examples for a pair of test images representing an agricultural area in summer and winter seasons. In this case, quasi-periodic field structures are dominant, and the number of false but plausible matches can be large. In the first example, the false matches are spatially separated, and a large search zone can be split into tiles containing a single match. This is not the case in the second example where the false matches are spatially close to each other. Under these conditions, the ability of DLSMarea to detect multiple matches at once is most useful. The last example comes from another pair of images representing the radar-to optical case. In this case, similarity for the true match is lower than in the optical-to-optical case and it is possible that false matches are more likely than the true one.

### 3.5. Localization Accuracy

In the next experiment, our goal is to verify the ability of DLSMarea to localize the true correspondence. To calculate the translation error, we use all the 30,000 test pairs that correspond to the true match. For each pair, a random subpixel shift in the range −3…3 pixels is applied to the RI fragment. This shift represents the ground truth value. For each SM version, the SM map is computed and used to find the position of the true match. In order to avoid the influence of false matches, only a small SM map fragment in the range −5…5 pixels is used. This fragment is guaranteed to cover the true correspondence position. The true correspondence position for the DLSMarea is estimated with subpixel accuracy according to the procedure proposed for the original DLSM model in [31].

All the 30,000 pairs are sorted in decreasing order of similarity according to the SM value of the detected true match. The localization error SD is calculated for successive groups of 500 patches.

If the position of the true match is found to be pixel accurate, the best achievable error SD corresponds to the SD of a uniform distribution in the interval [−0.5, 0.5] pixels equal to 0.2887. We measured the quality of the SM localization by the number of pairs with a localization SD below this threshold. The number of pairs localized with subpixel accuracy for the original DLSM and DLSMarea variants is detailed in Table 3.

Since DLSMarea SM inherits the design of the original DLSM, we only need to show that the localization accuracy of DLSMarea is not lower than that of DLSM. According to the data in Table 3, the original DLSM localizes 1440 pairs with subpixel accuracy, and DLSMarea localizes from 1463 to 2495 pairs depending on the $n_{s}$ value. We conclude that the localization accuracy of DLSMarea is not worse than that of the original DLSM SM.

## 4. Discussion

This paper addresses the problem of finding correspondences between multimodal images using a deep learning approach. In contrast to existing methods, we proposed a CNN that inputs a template fragment and a reference fragment enlarged to cover a search zone (e.g., ±16 pixels) and outputs an SM value for the entire search zone. The distinctive feature of the developed SM is that it detects not only the true correspondence but also other plausible matches in the search zone.

The training of the proposed CNN is complicated by the impossibility of annotating false matches between two images in a search zone. Only one—the true one—correspondence can be used as GT during training. To overcome this problem, we proposed a loss function that enforces the translation invariance of the DLSMarea output, i.e., the partial SM maps provided by the SM covering different parts of a larger search zone should coincide in the overlapping area. We demonstrated that this loss term also enables the detection of false putative matches in addition to the true correspondence.

The proposed DLSMarea predicts an SM map in a search zone (e.g., with the possible linear size of 17, 25, 33, 41, 49, 57, …, pixels). From these SM maps, multiple matches can be extracted. Each match is characterized by its SM value, its estimated position with subpixel accuracy and the covariance matrix of the position estimation error. The benefit of the designed SM is that it is computationally efficient (it processes the entire search zone at once), has high discriminative power in the multimodal case and detects multiple matches. The SM value of each match does not depend on the number and composition of other matches, and the SM map can be tiled to cover a wider search zone.

One limitation of the proposed method is that it is designed for searching along translation dimensions and does not support large rotation and scale errors (but it can tolerate small errors by using data augmentation during training). As mentioned above, this limitation is acceptable for remote sensing images where rotation and scale errors are compensated for by using the orbital parameters of the sensors.

One of the main applications of the proposed “area” SM is multiple match registration of complex multimodal RS images, including images with repetitive structures, high noise levels or large structural differences (e.g., optical-to-DEM registration). We leave this interesting problem for future work.

## Figures and Tables

**Table 1:** Components of the loss function.

Requirement	$L_{\mathrm{main}.\mathrm{peak}}$	$L_{\mathrm{discrimination}}$	$L_{\mathrm{shift}}$	$L_{\mathrm{rotation}}$
Translation invariance	-	-	+	-
Detection of multiple matches	-	-	+	-
Subpixel accuracy of the main lobe localization without the need for intensity or SM interpolation	+	-	-	-
Estimation of the accuracy of lobe localization, including anisotropic case	+	-	-	+
Discrimination between the main and false matches	-	+	-	-

**Table 2:** Comparison of AUC values for “point” and “area” SM version. The best AUC value in each column is marked in bold font.

Method	General	Optical-to-DEM	Optical-to-Optical	Optical-to-Radar	Radar-to-DEM
NCC	61.50	54.54	59.57	70.18	62.12
MI	59.23	57.05	68.40	63.84	54.92
SIFT-OCT	65.69	59.13	65.79	73.60	67.71
MIND	72.56	68.88	85.37	70.98	64.52
DLSM	83.86	80.00	88.49	83.23	81.95
DLSMarea17	84.39	82.10	90.14	80.20	82.90
DLSMarea25	86.37	**84.23**	91.98	83.17	85.85
DLSMarea33	**86.87**	83.98	**92.41**	**84.58**	**87.81**
DLSMarea41	84.32	80.79	88.81	80.43	86.86
DLSMarea49	86.05	83.10	91.89	83.11	86.42
DLSMarea57	85.14	84.05	90.70	82.30	83.61

**Table 3:** Comparison of AUC values for the “point” and the “area” SM version.

Method	Number of Pairs Localized with Subpixel Accuracy
DLSM	1440
DLSMarea17	2227
DLSMarea25	2324
DLSMarea33	2292
DLSMarea41	1463
DLSMarea49	2021
DLSMarea57	2495

## Data Availability

Not applicable.
